# Pediatric Inflammatory Bowel Disease with Cytoplasmic Staining of Antineutrophil Cytoplasmic Antibodies

**DOI:** 10.1155/2013/196012

**Published:** 2013-02-14

**Authors:** Omar I. Saadah, Jamil A. Al-Mughales

**Affiliations:** ^1^Division of Pediatric Gastroenterology, Department of Pediatrics, Faculty of Medicine, King Abdulaziz University, P.O. Box 80215, Jeddah 21589, Saudi Arabia; ^2^Department of Microbiology and Immunology, Faculty of Medicine, King Abdulaziz University, P.O. Box 80215, Jeddah 21589, Saudi Arabia

## Abstract

*Background*. It is unusual for the antineutrophil cytoplasmic antibody with cytoplasmic pattern (cANCA) to present in patients with inflammatory bowel disease (IBD) without vasculitis. The purpose of this study was to describe the occurrence and characteristics of pediatrics IBD with cANCA. *Methods*. A retrospective review of pediatric IBD associated with cANCA serology in patients from King Abdulaziz University Hospital, Saudi Arabia, between September 2002 and February 2012. *Results*. Out of 131 patients with IBD screened for cANCAs, cANCA was positive in 7 (5.3%) patients of whom 4 had ulcerative colitis and 3 had Crohn's disease. The median age was 8.8 years (2–14.8 years). Six (86%) were males. Of the 7 patients, 5 (71%) were Saudi Arabians and 2 were of Indian ethnicity. The most common symptoms were diarrhea, abdominal pain, weight loss, and rectal bleeding. None had family history or clinical features suggestive of vasculitis involving renal and respiratory systems. No difference in the disease location or severity was observed between cANCA positive and cANCA negative patients apart from male preponderance in cANCA positive patients. *Conclusion*. The occurrence of cANCA in pediatric IBD is rare. Apart from male preponderance, there were no peculiar characteristics for the cANCA positive patients.

## 1. Introduction

Perinuclear antineutrophil cytoplasmic antibodies (pANCAs) have been reported in patients with inflammatory bowel disease (IBD). The pANCA pattern has been associated with 40–80% of adults with ulcerative colitis (UC) and 5–25% of patients with Crohn's disease (CD) [1–3]. The pANCAs are directed against cytoplasmic constituents of neutrophils with a perinuclear staining pattern [4]. pANCA reactivity found in IBD differs from the classical ANCA with cytoplasmic staining pattern (cANCA) that was linked to the inflammatory activity of primary small vessels vasculitis. The primary antigen specificity for cANCA is serine proteinase-3 (PR-3) and for pANCA is myeloperoxidase (MPO) [3]. cANCA was reported rarely in young adults with IBD [5]. In this study, our aim was to report the occurrence of cANCA and to describe the clinical characteristics of children with cANCA positive IBD.

## 2. Patients and Methods

This is a retrospective chart and laboratory data review of children and adolescents with the diagnosis of IBD who were seen at the pediatric gastroenterology clinic at King Abdulaziz University Hospital, Jeddah, Saudi Arabia, between the time period of September 2002 and February 2012.

The study was approved by the Bioethical and Research Committee at the Faculty of Medicine at King Abdulaziz University, and the study was conducted according to the principles of Helsinki Declaration.

The patients were diagnosed as UC and CD according to the international criteria established by the working groups of the European Society for Pediatric Gastroenterology, Hepatology and Nutrition (ESPGHAN) and North American Society for Pediatric Gastroenterology, Hepatology and Nutrition (NASPGHAN) based on a combination of clinical, laboratory, imaging, endoscopic, and histopathology features [6, 7]. The extent of the disease in UC patients was classified based on Montreal classification into distal colitis (mucosal changes limited to the rectum and sigmoid), left-sided colitis (mucosal changes up to the splenic flexure), or extensive colitis (mucosal changes beyond the splenic flexure) [8]. The disease phenotype in CD was classified according to Vienna classification into small bowel disease (L1), isolated colonic disease (L2), and ileocolonic disease (L3) [9]. For patients with UC, endoscopic assessment was graded on a scale of normal (score 0), mild disease (erythema, decreased vascular pattern, and mild friability) (score 1), moderate disease (marked erythema, lack of vascular pattern, friability, and erosions) (score 2), or severe disease (spontaneous bleeding and ulceration) (score 3) according to Mayo UC endoscopic score [10].

Testing for ANCAs in our laboratory is according to the recommendation by the International Consensus Statement [11]. All samples should be tested first by Indirect Immunoflurescence (IFF) on ethanol-fixed human neutrophils. Only positive results are investigated with antigen-specific Elisa assay. Because some antinuclear antibodies (ANA) may also react with nuclei of ethanol-fixed human neutrophils, before reporting the result of ANCA, we usually examine the patient serum for anti-nuclear antibodies (ANAs) in addition to testing patient samples on human neutrophils fixed in formalin instead of ethanol. IFF testing on ethanol-fixed human neutrophils usually run as recommended by the manufacturer (INOVA, USA). All the substrate slides for ANCA were reached to room temperature prior to removal from its pouch and labelled with pencil and placed in a suitable moist chamber. Then one drop (20–25 *μ*L) of the undiluted positive and the negative control were added to wells 1 and 2, respectively. The diluted (1 : 20) patient serum was added to the other wells. Slides were incubated for 30 minutes in a moist chamber then washed by a stream of PBs II buffer for 5 minutes. Fluorescent conjugate was added and incubated for 30 minutes, and then the slides were washed again as mentioned above and the slides were mounted by mounting medium and covered with cover slip for reading. Positive and negative cANCA or pANCA results were determined by brilliant apple green fluorescence under the Olympus fluorescent microscope ×40 as followed [12].

**Figure 1:**
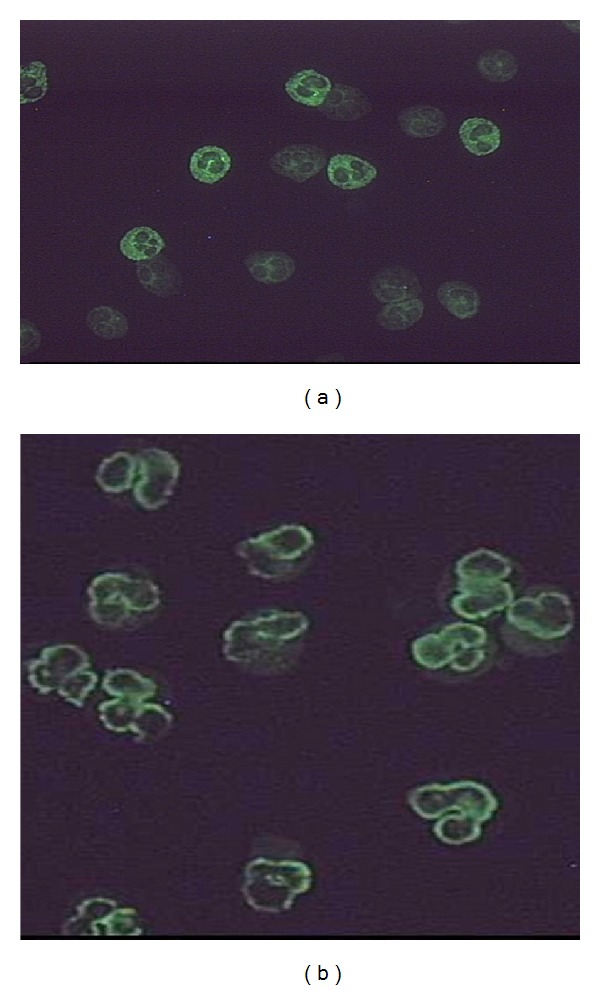
Pattern of immunofluorescence (IFF) staining on ethanol-fixed human neutrophils: (a) cytoplasmic staining pattern (cANCA); (b) perinuclear staining pattern (pANCA).

Two major staining patterns are shown from the IFF of ethanol-fixed neutrophils known as cANCA pattern that appears as granular cytoplasmic fluorescence with central interlobular accentuation (Figure 1(a)) and pANCA pattern that appears as localized staining just around the nucleus (Figure 1(b)). We routinely perform ANCA Elisa for antibodies to MPO and PR-3 (Quantite, INOVA Diagnostic, Inc) in all ANCA IFF positive sera. A cut-off value of 20 is taken as recommended by the manufacturer.

Statistical analyses were performed using SPSS 19 software (SPSS, Inc., Chicago, III). Data were expressed as percentage of the total for categorical variables, as mean with standard deviation (SD) for normally distributed continuous variables, or as median with interquartile range for skewed distributed variables. Group comparison of variables was performed by the nonparametric tests (Mann-Whitney) for continuous variables and chi square/Fisher's exact for categorical variables. *P* value less than 0.05 was considered significant.

## 3. Results

Out of 131 patients with IBD screened for cANCAs, it was positive in only 7 (5.3%) patients. The clinical characteristics of cANCA positive patients are outlined in Table 1. Four (57%) patients had UC, and 3 (43%) patients had CD. The median age was 8.8 years (range, 2–14.8). Six (86%) were males. Of the 7 patients, five (71.4%) were Saudi Arabians and 2 (28.6%) were of Indian ethnicity. The most common clinical symptoms were diarrhea, abdominal pain, weight loss, and rectal bleeding. The median duration of symptoms was 7 months (range, 5–24 months). None had clinical features or laboratory abnormalities suggestive of vasculitis involving skin, kidneys, or upper and lower respiratory systems. None had family history of IBD or vasculitis.

Laboratory investigations (Table 2) showed the mean ± SD hemoglobin of 9.5 ± 1.9 g/dL (normal, 12–14.5 g/dL), mean ± SD platelets count of 603 ± 148 k/uL (normal, 150–450 k/uL), mean ± SD albumin level of 30.4 ± 5.5 g/L (normal, 35–50 g/L), and mean ± SD C-reactive protein (CRP) of 65.4 ± 51.8 mg/L ((normal, 0–3 mg/L).

All patients were positive for antibodies to PR-3, the putative antigen for cANCA in Wegener's granulomatosis by Elisa, but none was positive for MPO, the antigen for pANCA. ASCAs were positive in all CD patients.

Comparing cANCA positive with cANCA negative patients (Table 3), apart from significant male predominance in the cANCA positive group (*P* = 0.03), there was no difference in the mean age at presentation (*P* = 0.5) and in the phenotype or distribution of the disease in both UC and CD patients, respectively (*P* = 0.5 and *P* = 0.4). For patients with UC, the Mayo score did not differ between the cANCA positive and cANCA negative patients (*P* = 0.33). There was no difference either in the requirement for systemic corticosteroids or the need for colectomy (*P* = 0.46 and *P* = 0.73).

## 4. Discussion

In this study, seven patients with IBD in association with cANCAs were identified. This pattern usually resulted from antibodies to the 29 kDa serine protease-3 (PR-3) that were positive in all our patients but can also be seen in association with other neutrophil cytoplasmic enzymes [3]. The cANCA pattern is usually associated with Wegener's granulomatosis (WG) [13, 14] which is a form of necrotizing vasculitis that occurs mainly in Caucasian adults and involves the small- and medium-size blood vessels with formation of granulomata and commonly affects the respiratory, ocular, and renal systems [15]. The occurrence of WG in children is rare. Akikusa et al. [16] reported 25 pediatric patients diagnosed over a 21-year period with WG that demonstrated female predominance in 4:1 ratio. Renal involvement occurs in 88%, upper airway involvement in 84%, and lung involvement in 80% of the patients. The absence of symptoms of respiratory, renal, or ocular involvement makes the diagnosis of WG unlikely in our patients.

Patients with both forms of IBD most frequently UC usually mount a response to atypical pANCAs but not to cANCAs that commonly react to MPO rather than PR-3 antigen. It is unusual to detect cANCA in patients with IBD. However, Freeman [5] has reported 18 adult patients with IBD colitis with associated cANCAs. All were with extensive colitis and none had symptoms suggestive of WG. Four patients with ulcerative colitis and cANCA had an extensive colitis, and no patients had less extensive disease keeping with the report by Freeman. Furthermore, we had two patients with small bowel involvement that was not seen in patients reported by Freeman. The cANCA positive patients in Freeman report constituted less than 2% of the total IBD patients. Our report is the first series of pediatric IBD associated with positive cANCA that constituted 5.3% of our IBD patients screened for cANCA. For unclear reason, we found more affected males than females, an observation that was not seen in Freeman series.

The gastrointestinal involvement in WG was reported very rarely [17, 18] and occasionally complicated by intestinal perforation [19, 20]. However, the clinical picture in the presence of gastrointestinal involvement is always dominated by the symptoms due to respiratory and renal involvement.

Interestingly, two of the seven patients in our report were of Indian origin, an observation that was also made by Freeman who reported that 7 out of 18 patients were of Indian ethnicity. This may suggest an ethnic basis that may influence the tendency to mount response of cANCAs.

The implication of cANCA reactivity in our pediatric patients with IBD is not clear, as we found no difference in the age at presentation, disease localization, and severity, or requirement for treatment with corticosteroids or the need for colectomy between cANCA positive and cANCA negative patients.

In conclusion, we described a case series of IBD pediatric patients with the rare association with cANCA antibodies in the absence of evidence for systemic or local vasculitis. Apart from male predominance, no difference was observed in terms of disease presentation and outcome between cANCA positive and cANCA negative patients.

## Figures and Tables

**Table 1:** Characteristics of IBD patients with positive c-ANCA.

Patient	Age (years)	Sex	Nationality	Symptoms	Perianal disease/fistula	Diagnosis	Phenotype/extent	Extraintestinal manifestations	Mayo score
Patient 1	13.2	Male	Saudi	Diarrhea, abdominal pain, weight loss	No	CD	Ileocolonic	Arthralgia	NA
Patient 2	8.9	Male	Indian	Diarrhea, abdominal pain, weight loss	Yes	CD	Ileocolonic	No	NA
Patient 3	14.8	Male	Saudi	Diarrhea, abdominal pain, weight loss, fever, perianal disease, fistula	Yes	CD	Colonic	No	NA
Patient 4	6.4	Male	Indian	Diarrhea, rectal bleeding, abdominal pain	No	UC	Extensive	No	2
Patient 5	2	Male	Saudi	Diarrhea, rectal bleeding, abdominal pain	No	UC	Extensive	No	3
Patient 6	6	Female	Saudi	Diarrhea, rectal bleeding, abdominal pain	No	UC	Extensive	No	3
Patient 7	8.8	Male	Saudi	Diarrhea, rectal bleeding	No	UC	Extensive	No	3

CD: Crohn's disease, UC: ulcerative colitis, and NA: not applicable.

**Table 2:** Laboratory investigations of IBD patients with positive c-ANCA.

Patient	Hb (g/dL)	Platelets k/uL	Albumin (g/L)	CRP mg/L	ALT (IU)	ASCA-A	ASCA-G	Anti-MPO	Anti-PR3
Patient 1	9.4	731	32	148	23	Neg.	Pos.	Neg.	Pos.
Patient 2	12	655	36	120	37	Pos.	Neg.	Neg.	Pos.
Patient 3	9.6	650	22	30	21	Pos.	Neg.	Neg.	Pos.
Patient 4	10	381	43	16	35	Neg.	Neg.	Neg.	Pos.
Patient 5	6	785	24	80	22	Neg.	Neg.	Neg.	Pos.
Patient 6	8.3	587	36	41	41	Neg.	Neg.	Neg.	Pos.
Patient 7	11.2	434	33	23	23	Neg.	Neg.	Neg.	Pos.

Hb: hemoglobin, CRP: c-reactive protein, ALT: alanine aminotransferase, ASCA-A: IgA anti-*Saccharomyces cerivisiae* antibody, ASCA-G: IgG anti-*Saccharomyces cerivisiae* antibody, anti-MPO: myeloperoxidase antibody, anti-PR-3: serine proteinase-3 antibody, Pos.: positive, and Neg.: negative.

**Table 3:** Comparison of cANCA positive with cANCA negative patients.

	cANCA positive patients (*n* = 7)	cANCA negative patients (*n* = 124)	*P* value**
Age (years), mean ± SD	8.6 ± 4.3	10.1 ± 5.2	0.5*
Male:female	6:1	54:70	0.03
Diagnosis			
Ulcerative colitis (*n* = 45)	4	41	0.19
Crohn's disease (*n* = 87)	3	83	
Disease extension (UC)			
Rectosigmoid	0	6	
Left sided	0	5	0.26
Extensive	4	30	
Mayo score (UC)			
Score 2	1	16	0.33
Score 3	3	25	
Disease phenotype (CD)			
Colonic	1	25	
Ileal	0	25	0.5
Ileocolonic	2	34	
Steroid treatment	7	115	0.46
Colectomy	0	2	0.73

**Chi square; *Mann-Whitney *U* test.
